# Older age should not be a barrier to testing for somatic variants in homologous recombination DNA repair-related genes in patients with high-grade serous ovarian carcinoma

**DOI:** 10.1016/j.tranon.2023.101638

**Published:** 2023-02-18

**Authors:** Omali Pitiyarachchi, Yeh Chen Lee, Hao-Wen Sim, Sivatharsny Srirangan, Cristina Mapagu, Judy Kirk, Paul R. Harnett, Rosemary L. Balleine, David D.L. Bowtell, Goli Samimi, Alison H. Brand, Deborah J. Marsh, Philip Beale, Lyndal Anderson, Natalie Bouantoun, Pamela Provan, for the INOVATe Investigators, Susan J. Ramus, Anna DeFazio, Michael Friedlander

**Affiliations:** a School of Biomedical Sciences, Faculty of Medicine and Health, UNSW Sydney, Sydney, NSW, Australia; b School of Clinical Medicine, Faculty of Medicine and Health, UNSW Sydney, Sydney, NSW, Australia; c Department of Medical Oncology, Prince of Wales and Royal Hospital for Women, Randwick, NSW, Australia; d NHMRC Clinical Trials Centre, The University of Sydney, Camperdown, NSW, Australia; e Department of Medical Oncology, The Kinghorn Cancer Centre, Darlinghurst, NSW, Australia; f Department of Medical Oncology, Chris O'Brien Lifehouse, Camperdown, NSW, Australia; g Garvan Institute of Medical Research, Sydney, NSW, Australia; h Centre for Cancer Research, Westmead Institute for Medical Research, Westmead, NSW, Australia; i Department of Gynaecological Oncology, Westmead Hospital, Westmead, NSW, Australia; j Faculty of Medicine and Health, The University of Sydney, Sydney, NSW, Australia; k Illawarra and Shoalhaven Cancer Care Centres, Wollongong and Nowra, NSW, Australia; l The Crown Princess Mary Cancer Centre, Westmead Hospital, Westmead, NSW, Australia; m NSW Health Pathology, NSW, Australia; n Research Division, Peter MacCallum Cancer Centre, Melbourne, Victoria, Australia; o Sir Peter MacCallum Cancer Centre Department of Oncology, University of Melbourne, Parkville, Victoria, Australia; p National Cancer Institute, Bethesda, Maryland, United States of America; q Translational Oncology Group, School of Life Sciences, Faculty of Science, University of Technology Sydney, NSW, Australia; r Northern Clinical School, Faculty of Medicine and Health, The University of Sydney, Sydney, NSW, Australia; s Department of Tissue Pathology and Diagnostic Oncology, Royal Prince Alfred Hospital, Camperdown, NSW, Australia; t Adult Cancer Program, Lowy Cancer Research Centre, UNSW Sydney, Sydney, NSW, Australia; u The Daffodil Centre, The University of Sydney, a joint venture with Cancer Council NSW, Sydney, NSW, Australia

**Keywords:** Older patients, Somatic testing, Ovarian cancer

## Abstract

**Background:**

Somatic pathogenic variants (PVs) in homologous recombination DNA repair (HR)-related genes found in high-grade serous ovarian carcinomas (HGSC) are not well-characterised in older patients (≥70 years). This may reflect low testing rates in older patients.

**Methods:**

Data from 1210 HGSC patients in AACR Project GENIE and 324 patients in an independent dataset INOVATe were analysed. Cases where somatic variants could be distinguished from germline variants were included, and analysis was restricted to those with a somatic *TP53* variant, to ensure cases were HGSC.

**Results:**

Of 1210 patients in GENIE, 27% (*n* = 325) were aged ≥70 years at testing. Patients with somatic-only PVs in *BRCA2* were older compared with *BRCA1* (median 71 vs 60 years, *p* = 0.002). Median age for 21 patients with somatic-only PVs in 11 other HR-related genes ranged from 40 to 67 years. In older patients, 7% (*n* = 22) had somatic *BRCA1/2* PVs, and 1% (*n* = 2) had PVs other HR-related genes; this rate was not significantly different to younger patients (<70 years), 7% (*n* = 62) *BRCA1/2* and 2% (*n* = 19) other HR-related genes (*p* = 0.36). The overall frequency of somatic *BRCA1/2* PVs was similar in INOVATe (*n* = 25; 7.7%) and somatic-only *BRCA2* PVs were again found in older patients compared with *BRCA1* (median age: at testing, 70 vs 63 years; at diagnosis, 68 vs 60 years)*.*

**Conclusions:**

The overall frequency of somatic-only PVs in HR-related genes was similar in older and younger patients with HGSC, highlighting the importance of somatic testing irrespective of age. Limiting somatic testing by age may exclude patients who could benefit from maintenance poly(ADP-ribose) polymerase (PARP) inhibitors.

## Highlights

• 7% somatic *BRCA1/2* pathogenic variant (PV) rate in older high-grade serous ovarian carcinoma (HGSC) patients.
• Overall somatic PV frequency in 13 homologous recombination DNA repair-related genes was similar in older and younger patients.
• Somatic *BRCA2* PVs occurred in older patients (median age 71).
• Excluding older patients from testing may miss a therapeutic opportunity.

## Graphical abstract

**Figure:**
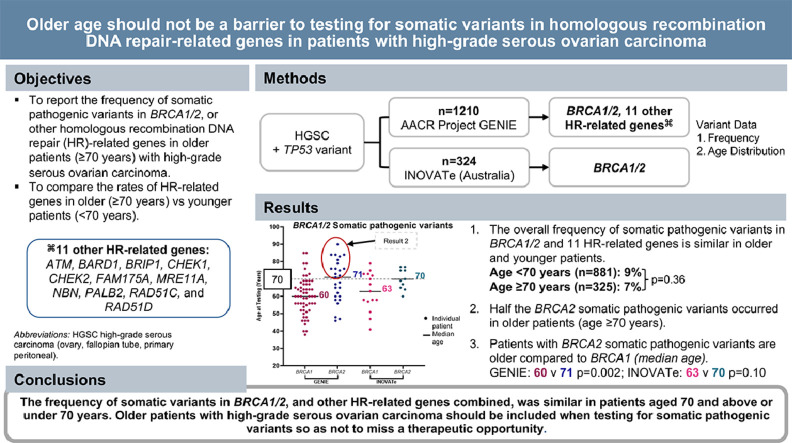

## Introduction

High-grade serous carcinoma (HGSC) is the most common subtype of epithelial ovarian cancer (EOC, including fallopian tube and peritoneal cancer). The mean age at diagnosis is 61.2 years [1], but almost 40% of patients are over the age of 70 at initial presentation [2]. There is an increasing proportion of older adults in the population, with 15–20% of people in countries such as the USA and Australia aged 65 and over [3], yet older patients are under-represented in oncology clinical trials, and tend to have worse outcomes [4], [5], [6]. Most older patients with EOC have advanced stage disease at diagnosis, but receive less treatment, including maintenance therapies, compared to younger patients [2]. This is not explained entirely by patient comorbidities [7,8].

Contemporary management of patients with HGSC includes cytoreductive surgery and platinum-based chemotherapy, with maintenance poly (ADP-ribose) polymerase inhibitors (PARPi) [9,10] with or without bevacizumab [11] in a selected subset of patients. In the first line setting the American Society of Clinical Oncology (ASCO) [12] and European Society for Medical Oncology (ESMO) [13] recommend maintenance PARPi after a complete or partial response to platinum-based chemotherapy in patients with advanced EOC, with olaparib for those with a germline or somatic *BRCA1/2* pathogenic variant (PV) or likely PV, or niraparib for those without an identified *BRCA1/2* PV [9,10]. In patients with recurrent EOC, guidelines recommend either olaparib, niraparib or rucaparib as maintenance therapy following response to platinum-based chemotherapy for patients who have not received a PARPi previously, regardless of *BRCA1/2* PV status [14,15]. However, a consistent finding in all trials in both first line and beyond is a longer progression free survival (PFS) in patients with a germline or somatic *BRCA1/2* PV, followed by BRCA wild-type/homologous recombination DNA repair (HR) deficient cancers, compared to patients with HR proficient HGSC [9,11]. In some countries, such as Australia, access to maintenance PARPi is restricted to patients with either a germline or somatic *BRCA1/2* PV, where it is available as part of the universal healthcare scheme. However, even in countries such as the USA with broad approvals for maintenance therapy in patients with advanced stage EOC, recent data indicate that only 31% of patients receive front-line maintenance PARPi [16], and the reasons for low uptake and the barriers to treatment are unclear.

Germline *BRCA1/2* PVs are found in 13–21% [17], [18], [19] of patients with HGSC, and are present in a higher frequency in younger patients [17,20]. For example, Alsop et al. [17] reported a germline *BRCA1/2* PV in 24% of patients between the ages 41–50,17% between the ages 51–60, and 8.3% in patients aged 65–80. Similar age-related frequencies were reported by Zhang et al. in unselected EOC patients [20]. The frequency of germline PVs has been reported to be as low as 1% (1/86) in women with EOC aged ≥70 years in an unselected population from the UK GTEOC study [21]. The age-related frequency of somatic-only *BRCA1/2* PVs were not reported in these studies.

Somatic *BRCA1/2* PVs are found in 6–11% of HGSC [18,19,22], with the age-related frequency not well reported. Patients with somatic *BRCA1/2* PVs derive a similar benefit to PARPi as patients with germline *BRCA1/2* PVs [23]. This underscores the importance of somatic *BRCA1/2* testing particularly if HR deficiency (HRD) testing is not available or affordable [9,24,25]. Moreover, somatic PVs in a number of other genes related to HRD may also be associated with response to a PARPi [19,26].

The American Society of Clinical Oncology (ASCO) [27] recommends germline genetic testing for *BRCA1/2* and ‘other ovarian cancer susceptibility genes’ for all women diagnosed with EOC. Where a germline PV or likely PV is not found, somatic testing of the tumour for *BRCA1/2* is recommended. However, real-world data shows that rates of germline testing are variable with reports of only 10 to 55% of patients being tested [28], [29], [30]. Various factors can influence testing rates including the treating centre and patient insurance status [28]. Large registry data suggests the rate of testing for germline PVs for women with ovarian cancer in the US overall is in the order of 30% [31], with Cham et al. 2022 [32] showing lower testing rates (18%) in women aged ≥65 years. The proportion of older patients who undergo somatic testing is likely to be lower, as was demonstrated by Huang et al. in a series of 367 women with EOC where the overall rate of germline testing was 55% and 27% received somatic testing [28].

Somatic PVs and other forms of DNA damage accumulate with age [33], [34], [35], [36]. The frequency of somatic PVs in HR-related genes including *BRCA1/2* in older patients with HGSC is not well reported. We hypothesised that the rate of somatic PVs of the selected genes would increase with age. To investigate this, we examined the age distribution of somatic PVs in *BRCA1/2* and 11 HR-related genes (*ATM, BARD1, BRIP1, CHEK1, CHEK2, FAM175A, MRE11, NBN, PALB2, RAD51C, RAD51D*) [19] in patients with HGSC using publicly available datasets. We also utilised data from the prospective precision oncology study INOVATe (Individualised Ovarian Cancer Treatment Through Integration of Genomic Pathology into Multidisciplinary Care), which is a multicentre collaboration involving 12 sites in Sydney, Australia providing tumour genomic profiling for patients with ovarian cancer. We selected ≥70 years of age as data suggests a relatively low frequency of somatic testing in older patients [37] and it is commonly used as the age cut-off for germline testing in patients with no family history of breast/ovarian cancer [38].

## Methods

The selection of genes for our analysis is based on the publication by Pennington et al. [19] which reported the frequency of *BRCA1/2* and 11 HR-related genes (*ATM, BARD1, BRIP1, CHEK1, CHEK2, FAM175A, MRE11A, NBN, PALB2, RAD51C*, and *RAD51D)* in patients with EOC, and found the presence of a germline or somatic PV in any of these genes were associated with response to platinum chemotherapy and overall survival.

### GENIE

The AACR Project GENIE cohort version 11.0-public database represents clinical-grade genomic sequencing data generated and collated from 19 institutions in the USA, accessed using cBioPortal [39,40]. To differentiate somatic from germline variants, results from both tumour and germline were required for our analysis and review of the GENIE 11.0-public data guide [41] showed that sequencing from the Memorial Sloan Kettering Cancer Centre (MSKCC) met this requirement. Sequencing had been performed on both tumour and normal samples, and germline PVs were excluded leaving only somatic variants for analysis [42]. Tumour profiling had been performed on formalin-fixed paraffin-embedded (FFPE) tumour specimens and patient-matched normal samples using the custom panels MSK-IMPACT 505, MSK-IMPACT 468, MSK-IMPACT 410 and MSK-IMPACT 341. These custom panels comprised of all protein-coding exons of 505, 468, 410 and 341 cancer-associated somatic genes respectively. Point mutations/single-nucleotide variants (SNVs) and short indels which could be detected with commercial panel testing were included in this analysis. We only included predicted protein truncating or known missense variants in *BRCA1/2.* For the other HR-related genes, we only included those predicted as deleterious by the American College of Medical Genetics and Genomics (ACMG) and the Association for Molecular Pathology (AMP) variant classification guidelines [43].

**Fig. 1:**
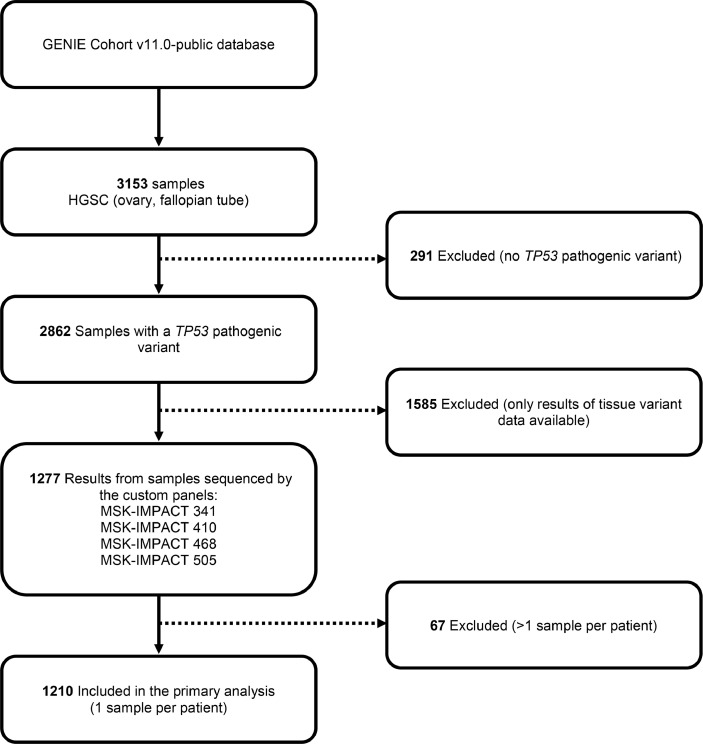
Process of sample selection from AACR Project GENIE Cohort v11.0-public database .

Progressive selection criteria were applied in cBioPortal to identify patients with HGSC, to select only tumours with a somatic *TP53* variant (to exclude cases that were not HGSC), and who had sequencing using the custom panels MSK-IMPACT-341, 410, 468 and 505 [see Fig. 1]. Cases with one tumour sample per patient were selected to preclude a potential situation where there may be cases with differing results in samples. Variant data from these samples were reviewed and fusions and copy number alterations were not included. The resulting variant data was analysed by patient age at reporting. *(Data last accessed 23rd February 2022)*.

### INOVATe cohort

Patients were recruited to the INOVATe study between 2016 – 2020 from 12 treatment centres in New South Wales, Australia under a protocol approved by the Western Sydney Local Health District Human Research Ethics Committee (4314 – 2019/ETH0190). Patients were included in this analysis if they had HGSC (ovarian, fallopian tube or primary peritoneal cancer) with a somatic *TP53* alteration. From this selection, the rate of variants in *BRCA1* or *BRCA2* was determined. Variants in *BRCA1* or *BRCA2* were included if they were detected in a tumour sample that was not found in a matched germline sample (by clinical genetic testing). *TP53* and *BRCA1/2* variants were detected amongst 30 genes on a custom ovarian cancer gene panel (QIAseq Custom Targeted DNA Panel; Qiagen, Clayton, Victoria, Australia) [44]. Library preparation and multi-gene sequencing was performed on DNA isolated from fixed or frozen tumour tissue as per the Qiagen protocol for ultrasensitive variant detection using integrated unique molecular indices (UMIs). Uniquely indexed samples were pooled and sequenced on an Illumina MiSeq V3 to generate 2 × 150 bp reads at a sequencing coverage of ∼x9000 reads per base. The NGS sequence analysis of the QIASeq Targeted DNA Custom multi-gene panel consisted of sequence alignment to human reference genome, build hg19/GRch37, and data analysis in accordance with smCounter2, a UMI-based variant caller [45]. Variants were curated in accordance with bioinformatics scores for SIFT and PolyPhen; followed by examination of ClinVar and dbSNP categorization. Genetic variants were interpreted according to ACMG/AMP guidelines [43,46]. In one case data was obtained from clinical tumour testing on a QIAseq targeted *BRCA1* and *BRCA2* DNA panel.

### Statistical analysis

The ages of patients with *BRCA1* versus *BRCA2* somatic PVs were compared using the Mann Whitney test. The age at diagnosis versus the age at testing per patient were compared using the paired *t*-test. Fisher's exact test was used to compare the proportion of patients who had a somatic PV in *BRCA1/2* or HR-related (versus those who did not) between those aged ≥70 years (versus <70 years). This was reported alongside the risk ratio of the presence of *BRCA1/2* or HR-related gene PV (95% confidence intervals computed using the Koopman asymptotic score) and the risk difference of the presence of *BRCA1/2* and HR-related gene PV (95% confidence intervals computed using the Newcombe/Wilson score with continuity correction). All analyses were carried out using GraphPad Prism 9.3.1.

## Results

Somatic predicted protein truncating variants in 13 HR-related genes were identified in a cohort of HGSC cases from MSKCC using data from GENIE Cohort v11.0-public database. There were 1210 samples from 1210 patients included in the final analysis (Fig. 1).

**Fig. 2:**
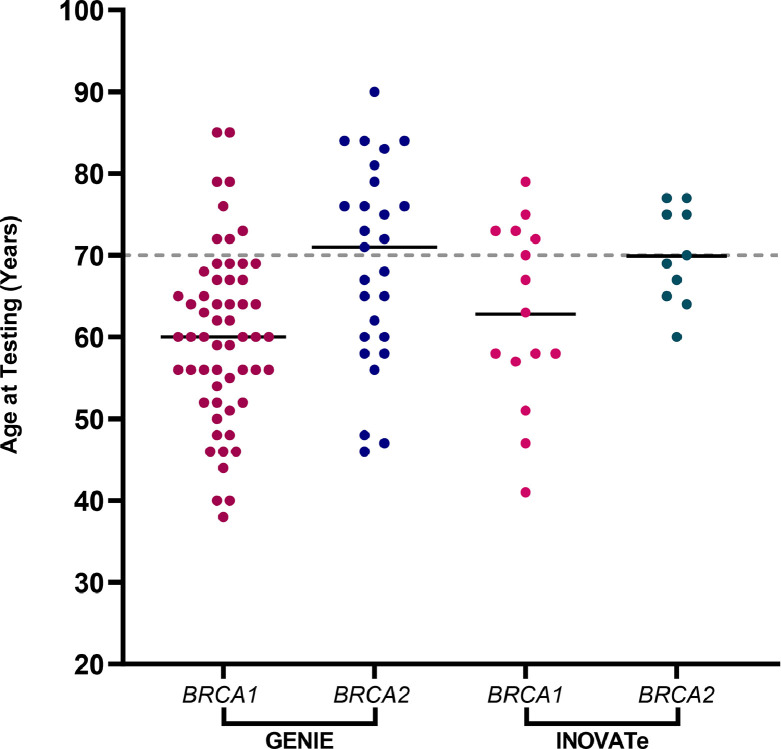
**Age distribution of *BRCA1/2* somatic-only pathogenic variants in GENIE and INOVATe.** Median age at testing of patient samples is represented by a horizontal line with patient samples represented by coloured dots. Dashed line denotes age 70 years.

Overall, somatic predicted protein truncating variants in *BRCA1/2* were found in 7% (*n* = 85) of tumours and 2% (*n* = 21) had a predicted protein truncating variant detected in one of the other 11 HR-related genes. Age was unknown for four patients, including one with a *BRCA1* PV. Older patients (≥70 years) comprised 27% (*n* = 325) of the total patient cohort. The frequency of somatic variants (Table 1) in patients aged ≥70 years was 7% (*n* = 22) for *BRCA1/2* and 1% (*n* = 2) for the 11 other HR-related genes combined and was not significantly different to those aged <70 years (i.e. somatic PV detected in 24 of 325 patients aged ≥70 years [7.38%] versus 81 of 881 patients aged <70 years [9.19%]; risk ratio of somatic PV status 0.80 [95% CI, 0.52 to 1.23]; risk difference of somatic PV status 1.81% [95% CI, −1.49% to 5.78%]; *p* = 0.36, Fisher's exact test). Patients with somatic *BRCA2* PVs were older compared with patients with *BRCA1* PVs (*p* = 0002, Mann-Whitney test). The median age of patients with a somatic *BRCA2* PV was 71 years in contrast to 60 years for *BRCA1* PV (Fig. 2). The median age of patients with somatic variants in the 11 other HR-related genes (*n* = 21) ranged from 40 to 67 years (Supplementary Table 1). Variant details are provided in Supplementary Table 2.

**Table 1:** GENIE – Number of patients with a somatic PV detected categorised by patient age.

	Patient Age^a^ (years)					

Genes	<49 (*n* = 116)	50–59 (*n* = 305)	60–69 (*n* = 460)	<70 Combined (*n* = 881)	≥70 (*n* = 325)	Somatic PV in age ≥70 as a proportion of all patients with a somatic PV^e^
*BRCA1*^b^	9	16	24	49	8	14%
*BRCA2*	3	3	7	13	14	52%
*PALB2* (& *ATM*^c^)			1	1		–
*ATM*	2	1	1	4	2	33%
*BRIP1*			3	3		–
*RAD51D*			1	1		–
*BARD1*	1			1		–
*NBN*		1	2	3		–
*CHEK1*	1			1		–
*CHEK2*		2		2		–
*MRE11*		1	1	2		–
*ABRAXAS1*			1	1		–
***BRCA1/2* Total**	12	19	31	62 (7%)	22 (7%)	26%
**Other 11 HR-related Genes Total**	4	5	10	19 (2%)	2 (1%)	10%
**Grand Total**	**16**	**24**	**41**	**81 (9%)**	**24 (7%)**	23%
				***p*** **=** **0.36**^d^		

a Age at which sequencing was reported.

b Age unknown (*n* = 4), including for a sample with *BRCA1* (*n* = 1).

c *ATM* missense variant also present.

d T-test used to compare the frequency of somatic PVs in patients ≥70 and <70 years.

e This column represents the number of patients aged ≥70 years with a somatic PV in that particular gene or selection of genes as a proportion of all patients with a somatic PV in that gene/selection of genes, represented as a percentage Blank cells indicate genes where somatic PVs were not detected; - represents cells where no percentage could be calculated as there were no patients aged ≥70 years with a somatic PV in that particular HR-related gene.

To further examine the finding that patients with somatic *BRCA2* PVs were older than those with somatic *BRCA1* PVs, we analysed tumour sequencing data from an independent patient cohort from the INOVATe study. Data from 333 HGSC patients were reviewed and those with a *TP53* alteration identified (*n* = 324) were selected for analysis. Both age at diagnosis and age at testing was available for this dataset. Thirty percent (*n* = 98) of patients diagnosed with HGSC were over the age of 70 years. Overall, the frequency of somatic *BRCA1* variants was 5% (*n* = 15) and *BRCA2* was 3% (*n* = 10). INOVATe patients with a *BRCA2* PV tended to be older with a median age at diagnosis of 68 years and 60 years for *BRCA1*, although the difference was not statistically significant in this smaller series (*p* = 0.11, Mann-Whitney test) (see Supplementary Table 3). Results were similar when age at testing was analysed, as a direct comparison with GENIE data. In INOVATe, the median age at testing *BRCA2* was 70 years, compared with 63 years for *BRCA1* (*p* = 0.10, Mann-Whitney test) (Fig. 2). The mean difference between age at diagnosis and age at testing for patients with a *BRCA1/2* PV was 2.7 years (range 0–10 years). Variant data is available in Supplementary Table 4.

## Discussion

In this analysis of sequencing data from 1210 patients with HGSC using AACR Project GENIE, the frequency of somatic only variants in HR-related genes in patients aged ≥70 years, was 7% which was similar to younger patients (aged <70 years). The majority of somatic PVs were in *BRCA1/2* with a minority in other HR-related genes. The median age of patients with a somatic *BRCA2* PV was 71 years in contrast to 60 years for in those with a somatic *BRCA1* PV. Sequencing results from the independent dataset INOVATe also supported the finding of somatic *BRCA2* PVs occurring in older patients. The INOVATe dataset also provided age at diagnosis, rather than age at reporting, which makes the results more generally applicable. Additionally, analysis of data from the GENIE cohort showed that if older age (≥70 years) was a barrier to testing, we would miss 52% of somatic *BRCA2* PVs and 23% of all somatic *BRCA*/HR-related gene PVs as these were present in patients aged ≥70 years.

The genes we analysed in GENIE were selected based on the publications by Pennington et al. 2014 [47] and Norquist et al. 2016 [48] which identified alterations in several non-*BRCA* HR-related genes in patients with EOC. Germline PVs in the HR-related genes *BRIP1, PALB2, RAD51C* and *RAD51D* have an established role in increasing a patient's risk of ovarian cancer [49], [50], [51]. Some of the genes in our selection have not been associated with increased risk of ovarian cancer (e.g.*CHEK1, CHEK2, MRE11, ABRAXAS11*), but we included them in our analysis as somatic variants in some of these genes may be associated with an increased sensitivity to PARPi [26,52], with further research being performed in this area.

The hypothesis for this study was based on the evidence that there is an age-related accumulation of cancer-associated somatic PVs in normal tissues [33,53,54]. *A priori* we postulated that there would also be an increase in the frequency of somatic variants in HR-related genes in older patients compared to younger patients. There is a paucity of data on the age-related frequency of somatic HR-related gene PVs in HGSC and this has clinical implications with respect to identifying patients who may benefit from maintenance therapy with a PARP inhibitor. Only a few studies have reported the frequency of these somatic PVs in relatively small numbers of older patients. In the AOCS/ICGC ovarian cancer dataset obtained from Patch et al. 2015, 2/14 (14%) patients with a somatic *BRCA1/2* PV were aged over 70 [22]. Of the 303 patients with HGSC and a *TP53* PV from the TCGA Nature 2011 dataset, 27 patients had a somatic PV identified in *BRCA1/2, ATM, PALB2* or *CHEK2*. Of these, 33% (9/27) were aged ≥70 years including 7 patients with *BRCA1/2,* 1 *ATM* and 1 *PALB2* PV [55]. Pennington et al. [47] published results from 390 unselected EOC patients who underwent germline and somatic testing. Of the patients with serous histology, 38% (5/13) patients with a somatic PV were aged ≥70 years of which all had a *BRCA1/2* variant. Other HR-related gene variants were noted in 3 younger patients, with a *BRIP1, CHEK2* or *RAD51C* variant respectively [47]. These studies suggested that there may be a larger proportion of somatic PVs identified in HR-related genes in patients ≥70 years with EOC. However, in our analysis with a larger number of patients we found a similar frequency of somatic PVs in HR-related genes in patients < 70 or ≥70 years of age. Potential reasons for not demonstrating an age-related increase includes the selection of this particular set of 13 genes in our analysis. It is possible that we may have missed other HR-related genes which are more common in the older cohort. Additionally, the population-based lower frequency of testing of older patients may have also had an impact.

There are some limitations on our analysis of the GENIE data. Although the data was collated from a large database, after application of our selection criteria and review of the AACR GENIE data guide [41], the results were derived from a single institution's contribution to the dataset. This is because MSKCC was the only contributor of results from HGSC samples with tumour and matched patient-normal sample profiling, and based on their methodology, we were confident our analysis was based on results of only somatic variants. The presence of a *TP53* pathogenic variant has been shown to be a defining feature of high grade serous ovarian carcinoma (HGSC), with the Cancer Genome Atlas database showing 96% of cases of HGSC harbouring a *TP53* pathogenic variant [56]. Subsequent review of the cases lacking a *TP53* pathogenic variant showed the majority were not HGSC [57]. As we were unable to review the histology from the cases sequenced using the MSK-IMPACT panel ourselves, we used the presence of a *TP53* pathogenic variant as part of our selection criteria to ensure the cases we included in our analysis were truly HGSC. For consistency, we also applied the same criteria to the INOVATe dataset.

We attempted to expand our dataset by exploring other publicly available databases but were limited by relatively low numbers of patients. The TCGA Nature 2011 dataset [55] sequenced 303 patients and identified 7% (*n* = 21) with a somatic *BRCA1/2* PV and 2% (*n* = 6) with an *ATM, PALB2* or *CHEK2* variant (see Supplementary Table 5). The AOCS/ICGC data set demonstrated 11% (*n* = 10) somatic *BRCA1/2* PVs in 92 patients [22] (see Supplementary Table 6), but these two datasets only comprised a small number of patients compared to the 1210 patients in the GENIE dataset. An additional limitation is that the GENIE cohort version 11.0-public database reports ‘age at which profiling of tumour specimen’ was performed. For the purpose of this analysis, we assumed this was close to the patient's age at diagnosis, as it is unlikely for there to be more than a few years discrepancy with age given the predicted survival of HGSC patients. However, we appreciate that some patients may have had sequencing performed at a later date such as at recurrence and not at initial diagnosis. Finally, by only including the 13 selected genes in our analysis it is possible that we may have missed other HR-related genes or included other genes that are not directly related to sensitivity to PARP inhibitors. To overcome some of these limitations, we analysed the independent dataset from INOVATe. As this dataset had a relatively smaller number of patients, results did not reach statistical significance, but comparison of the median ages does support the finding that somatic *BRCA2* PVs occurred more frequently in older patients compared with *BRCA1*. Additionally, we used the INOVATe data to calculate the mean difference between age at diagnosis and age at testing for the patients with a *BRCA1/2* PV which was 2.7 years, thus adding support to our assumption that age at testing is likely to be close to age at diagnosis in the GENIE dataset. Reversion mutations in *BRCA1/2* are more common than in the other HR-related genes and is associated with platinum resistance and exposure to PARP inhibitors. There is no data to suggest that reversion mutations differ by age, but rather related to the number of prior lines of platinum-based chemotherapy and PARP inhibitors [58]. In the INOVATe cohort, the result for the majority of samples tested 92% (23/25) were from a pre-chemotherapy tumour sample. In 2/25 cases the result was from a specimen obtained at interval debulking surgery i.e. these patients had received chemotherapy. In two separate cases we had both a pre-chemotherapy sample and a sample obtained at recurrence, with the same *BRCA* variant detected in the pre and post chemotherapy sample.

To capture a larger proportion of patients a broader screen for HRD status could be performed with commercially based FDA approved assays such as the myChoice HRD Plus assay (Myriad Genetic Laboratories) [59] or FoundationOne®CDx. Sabatier et al. [60] presented data from the PAOLA-1/ENGOT-ov25 first-line trial showing that older patients (≥65 years) with HRD-positive EOC who received olaparib and bevacizumab as upfront maintenance treatment had an improved PFS (HR 0.23; CI 0.14–0.39, *p*<0.0001) compared to those with HR-proficient EOC. The PFS benefit was similar in the younger patients (<65 years) with HRD-positive EOC who received the same treatment (HR 0.25; CI 0.18–0.36, *p*<0.0001). This is despite fewer older (≥65 years) patients showing positive tumour HRD status 35% (104/292) than the younger cohort (<65 years) 55% (283/514).

The ASCO [27] guideline “Germline and Somatic tumor Testing in Epithelial Ovarian Cancer” recommends all women diagnosed with EOC, regardless of family history, undergo germline testing with a panel that includes *BRCA1/2* and *RAD51C, RAD51D, BRIP1, MLH1, MSH2, MSH6, PMS2,* and *PALB2*. If germline testing is negative, the expert panel recommends proceeding to somatic tumour testing for *BRCA1/2*. The adoption of this recommendation has been impacted by several factors including cost and access to testing. As outlined by Chandrasekaran et al. [37], testing is sometimes limited to certain subsets of patients such as those with a specific histology (e.g. HGSC) or age (e.g. <70 years), thus resulting in low overall rates of somatic testing [31]. Access to testing varies internationally with some patients able to access it via universal healthcare programs and other patients requiring insurance or paying out-of-pocket. A USA population based study of 6001 patients from Surveillance, Epidemiology, and End Results (SEER) registry data showed that only 35% of women aged 70 and over diagnosed with EOC were undergoing germline genetic testing [31]. Limiting testing by age may exclude patients that could derive a therapeutic benefit from a PARPi, so it is pertinent to make clinicians aware not to limit testing to younger patients which was the main purpose of our study.

It is clear that further work needs to be done in expanding treatment options, such as offering maintenance therapy with PARPi to older patients with advanced EOC, particularly in those with germline or somatic PV in *BRCA1/2,* as these patients have the greatest benefit with treatment. Not only do older patients have a poor prognosis they make up over 40% of patients diagnosed with EOC [61], are underrepresented in clinical trials and may not receive optimal treatment even in the absence of comorbidities. Identifying somatic HR-related PVs in older patients with EOC, especially HGSC, may provide therapeutic options that could improve patient outcomes. Additional work is needed to provide evidence that alterations in any of these other HR-related genes are predictive of PARPi response in HGSC [52].

## Conclusion

The frequency of somatic-only PVs in HR-related genes was 7% (7% *BRCA1/2* and 1% other HR-related genes) in patients with HGSC aged ≥70 years. This is not significantly different to the frequency observed in somatic PVs in younger patients with HGSC. Patients with somatic *BRCA2* PVs are older than patients with somatic *BRCA1* PVs. If older patients were excluded from somatic testing, 52% of *BRCA2* PVs and 23% of all *BRCA*/HRD PVs would be missed. Therefore, older age should not be a barrier to testing, particularly for somatic *BRCA1/2* PVs given the potential benefit of maintenance PARPi which is independent of age.

## CRediT statement declaration

**Omali Pitiyarachchi:** Conceptualization; Data curation; Writing – original draft; Writing – review & editing; Visualization

**Yeh Chen Lee:** Conceptualization; Data curation; Writing – original draft; Writing – review & editing

**Hao-Wen Sim:** Formal analysis; Writing – review & editing

**Sivatharsny Srirangan:** Investigation; Writing – review & editing

**Cristina Mapagu:** Resources; Writing – review & editing

**Judy Kirk:** Data curation, Writing – review & editing

**Paul R. Harnett:** Resources; Writing – review & editing

**Rosemary L. Balleine:** Resources; Writing – review & editing

**David D. L. Bowtell:** Resources; Writing – review & editing

**Goli Samimi:** Resources; Writing – review & editing

**Alison H Brand:** Resources; Writing – review & editing

**Deborah J. Marsh:** Resources; Writing – review & editing

**Philip Beale:** Resources; Writing – review & editing

**Lyndal Anderson:** Resources; Writing – review & editing

**Natalie Bouantoun:** Resources; Writing – review & editing

**Pamela Provan:** Resources; Data curation; Writing – review & editing

**Susan J Ramus:** Conceptualization; Data curation; Writing – original draft; Writing – review & editing; Visualization; Supervision

**Anna DeFazio:** Conceptualization; Data curation; Writing – original draft; Writing – review & editing; Visualization; Supervision; Funding acquisition

**Michael Friedlander:** Conceptualization; Writing – original draft; Writing – review & editing; Visualization; Supervision

## Declaration of Competing Interest

The authors declare that they have no known competing financial interests or personal relationships that could have appeared to influence the work reported in this paper.

ADeF has received grant funding from AstraZeneca for unrelated work.

All other authors reported no conflicts of interest related to this work.

## Data Availability

Data will be made available on request.

## Appendix Supplementary materials

Image, application 1

## References

[1] Peres L.C. (2019). Invasive epithelial ovarian cancer survival by histotype and disease stage. J. Natl. Cancer Inst..

[2] Fourcadier E. (2015). Under-treatment of elderly patients with ovarian cancer: a population based study. BMC Cancer.

[3] United Nations, D., Population Division. World Population Prospects 2019. 2019 [cited 2021; Available from: http://population.un.org/wpp/.

[4] Sedrak M.S. (2021). Older adult participation in cancer clinical trials: a systematic review of barriers and interventions. CA-Cancer J. Clin.

[5] Scher K.S., Hurria A. (2012). Under-representation of older adults in cancer registration trials: known problem, little progress. J. Clin. Oncol..

[6] Hurria A. (2014). Designing therapeutic clinical trials for older and frail adults with cancer: U13 conference recommendations. J. Clin. Oncol..

[7] Jørgensen T.L. (2012). Significance of age and comorbidity on treatment modality, treatment adherence, and prognosis in elderly ovarian cancer patients. Gynecol. Oncol..

[8] Jordan S. (2013). Patterns of chemotherapy treatment for women with invasive epithelial ovarian cancer - a population-based study. Gynecol. Oncol..

[9] González-Martín A. (2019). Niraparib in patients with newly diagnosed advanced ovarian cancer. N. Engl. J. Med..

[10] Moore K. (2018). Maintenance olaparib in patients with newly diagnosed advanced ovarian cancer. N. Engl. J. Med..

[11] Ray-Coquard I. (2019). Olaparib plus bevacizumab as first-line maintenance in ovarian cancer. N. Engl. J. Med..

[12] Tew W.P. (2020). PARP Inhibitors in the Management of Ovarian Cancer: ASCO Guideline. J. Clin. Oncol..

[13] Colombo N., Ledermann J.A. (2021). Updated treatment recommendations for newly diagnosed epithelial ovarian carcinoma from the ESMO Clinical Practice Guidelines. Ann. Oncol..

[14] Mirza M.R. (2016). Niraparib Maintenance Therapy in Platinum-Sensitive, Recurrent Ovarian Cancer. N. Engl. J. Med..

[15] Pujade-Lauraine E. (2017). Olaparib tablets as maintenance therapy in patients with platinum-sensitive, relapsed ovarian cancer and a *BRCA1/2* mutation (SOLO2/ENGOT-Ov21): a double-blind, randomised, placebo-controlled, phase 3 trial. Lancet Oncol..

[16] Huelsmann E. (2022). Trends in frontline PARP inhibitor maintenance in advanced epithelial ovarian cancer across the United States. J. Clin. Oncol..

[17] Alsop K. (2012). BRCA mutation frequency and patterns of treatment response in BRCA mutation-positive women with ovarian cancer: a report from the Australian Ovarian Cancer Study Group. J. Clin. Oncol..

[18] (2011). The Cancer Genome Atlas Research Network, Integrated genomic analyses of ovarian carcinoma. Nature.

[19] Pennington K.P. (2014). Germline and somatic mutations in homologous recombination genes predict platinum response and survival in ovarian, fallopian tube, and peritoneal carcinomas. Clin. Cancer Res..

[20] Zhang S. (2011). Frequencies of BRCA1 and BRCA2 mutations among 1,342 unselected patients with invasive ovarian cancer. Gynecol. Oncol..

[21] Plaskocinska I. (2016). New paradigms for BRCA1/BRCA2 testing in women with ovarian cancer: results of the Genetic Testing in Epithelial Ovarian Cancer (GTEOC) study. J. Med. Genet..

[22] Patch A.-.M. (2015). Whole–genome characterization of chemoresistant ovarian cancer. Nature.

[23] Friedlander M. (2018). Long-term efficacy, tolerability and overall survival in patients with platinum-sensitive, recurrent high-grade serous ovarian cancer treated with maintenance olaparib capsules following response to chemotherapy. Br. J. Cancer.

[24] Faraoni I., Graziani G. (2018). Role of BRCA Mutations in Cancer Treatment with Poly(ADP-ribose) Polymerase (PARP) Inhibitors. Cancers (Basel).

[25] Mohyuddin G.R. (2020). Similar response rates and survival with PARP inhibitors for patients with solid tumors harboring somatic versus Germline BRCA mutations: a Meta-analysis and systematic review. BMC Cancer.

[26] Hodgson D.R. (2018). Candidate biomarkers of PARP inhibitor sensitivity in ovarian cancer beyond the BRCA genes. Br. J. Cancer.

[27] Konstantinopoulos P.A. (2020). Germline and somatic tumor testing in epithelial ovarian cancer: ASCO guideline. J. Clin. Oncol..

[28] Huang M. (2019). Identifying disparities in germline and somatic testing for ovarian cancer. Gynecol. Oncol..

[29] Childers C.P. (2017). National estimates of genetic testing in women with a history of breast or ovarian cancer. J. Clin. Oncol..

[30] Dewdney S. (2020). Low rates of BRCA1 and BRCA2 testing for patients with ovarian cancer in ASCO's CancerLinQ, a real-world database. J. Clin. Oncol..

[31] Kurian A.W. (2019). Genetic testing and results in a population-based cohort of breast cancer patients and ovarian cancer patients. J. Clin. Oncol..

[32] Cham S. (2022). Use of Germline BRCA testing in patients with ovarian cancer and commercial insurance. JAMA Netw. Open.

[33] Risques R.A., Kennedy S.R. (2018). Aging and the rise of somatic cancer-associated mutations in normal tissues. PLos Genet..

[34] Kennedy S.R., Loeb L.A., Herr A.J. (2012). Somatic mutations in aging, cancer and neurodegeneration. Mech. Ageing Dev..

[35] Vijg J., Dong X. (2020). Pathogenic mechanisms of somatic mutation and genome mosaicism in aging. Cell.

[36] López-Otín C. (2013). The hallmarks of aging. Cell.

[37] Chandrasekaran D. (2021). Implementation of multigene germline and parallel somatic genetic testing in epithelial ovarian cancer: SIGNPOST study. Cancers (Basel).

[38] Cancer Australia. Position Statement - Genetic testing for women diagnosed with ovarian cancer. 2017 20th January 2022]; Available from: https://www.canceraustralia.gov.au/system/tdf/position-statements/genetic_testing_for_women_diagnosed_with_ovarian_cancer.pdf?file=1&type=node&id=5436.

[39] Cerami E. (2012). The cBio cancer genomics portal: an open platform for exploring multidimensional cancer genomics data. Cancer Discov..

[40] Gao J. (2013). Integrative analysis of complex cancer genomics and clinical profiles using the cBioPortal. Sci. Signal.

[41] The AACR Project GENIE Consortium (2017). AACR Project GENIE: powering precision medicine through an International Consortium. Cancer Discov..

[42] Cheng D.T. (2015). Memorial sloan kettering-integrated mutation profiling of actionable cancer targets (MSK-IMPACT): a hybridization capture-based next-generation sequencing clinical assay for solid tumor molecular oncology. J. Mol. Diagn..

[43] Richards S. (2015). Standards and guidelines for the interpretation of sequence variants: a joint consensus recommendation of the American College of Medical Genetics and Genomics and the Association for Molecular Pathology. Genet. Med..

[44] QIAGEN. QIAseq Targeted DNA Panel Handbook. 2022 [cited 2022 August]; Available from: https://www.qiagen.com/au/resources/resourcedetail?id=8907edbe-a462-4883-ae1b-2759657e7fd0&lang=en.

[45] Xu C. (2019). smCounter2: an accurate low-frequency variant caller for targeted sequencing data with unique molecular identifiers. Bioinformatics.

[46] Li M.M. (2017). Standards and guidelines for the interpretation and reporting of sequence variants in cancer: a joint consensus recommendation of the Association for Molecular Pathology, American Society of Clinical Oncology, and College of American Pathologists. J. Mol. Diagn..

[47] Pennington K.P. (2014). Germline and somatic mutations in homologous recombination genes predict platinum response and survival in ovarian, fallopian tube, and peritoneal carcinomas. Clin. Cancer Res..

[48] Norquist B.M. (2016). Inherited mutations in women with ovarian carcinoma. JAMA Oncol..

[49] Song H. (2015). Contribution of germline mutations in the RAD51B, RAD51C, and RAD51D genes to ovarian cancer in the population. J. Clin. Oncol..

[50] Ramus S.J. (2015). Germline mutations in the BRIP1, BARD1, PALB2, and NBN genes in women with ovarian cancer. J. Natl. Cancer Inst..

[51] Tischkowitz M. (2021). Management of individuals with germline variants in PALB2: a clinical practice resource of the American College of Medical Genetics and Genomics (ACMG). Genet. Med..

[52] Lheureux S. (2017). Long-term responders on olaparib maintenance in high-grade serous ovarian cancer: clinical and molecular characterization. Clin. Cancer Res..

[53] Li R. (2021). A body map of somatic mutagenesis in morphologically normal human tissues. Nature.

[54] Moore L. (2021). The mutational landscape of human somatic and germline cells. Nature.

[55] Bell D. (2011). Integrated genomic analyses of ovarian carcinoma. Nature.

[56] Network T.C.G.A.R. (2011). Integrated genomic analyses of ovarian carcinoma. Nature.

[57] Vang R. (2016). Molecular alterations of TP53 are a defining feature of ovarian high-grade serous carcinoma: a rereview of cases lacking TP53 Mutations in The Cancer Genome Atlas Ovarian Study. Int. J. Gynecol. Pathol..

[58] Norquist B. (2011). Secondary Somatic Mutations Restoring BRCA1/2 Predict Chemotherapy Resistance in Hereditary Ovarian Carcinomas. J. Clin. Oncol..

[59] Myriad Genetic Laboratories, I. Myriad MyChoice® CDx Plus Technical Specifications. 2022 May 2022; Available from: https://myriad-library.s3.amazonaws.com/technical-specifications/myChoice+CDx+Plus+Technical+Specifications.pdf.

[60] Sabatier, R., et al., 739P Efficacy and safety of maintenance olaparib and bevacizumab (bev) in ovarian cancer (OC) patients (pts) aged ≥65 years (y) from the PAOLA-1/ENGOT-ov25 first-line trial. Ann. Oncol., 2021. 32: p. S737–S738.

[61] Tortorella L. (2017). Ovarian cancer management in the oldest old: improving outcomes and tailoring treatments. Aging Dis..

