# Supplementary material for: Older age should not be a barrier to testing for somatic variants in homologous recombination DNA repair-related genes in patients with high-grade serous ovarian carcinoma
Source: Transl Oncol. 2023 Feb 18;31:101638. doi: 10.1016/j.tranon.2023.101638 (PMC9971549; doi:10.1016/j.tranon.2023.101638)
Supplement: Supplementary file 1 [file mmc1.docx]

**Supplementary Material**

- **Supplementary Table 1. GENIE - Detailed age at reporting results of patients with somatic pathogenic variants.**
- **Supplementary Table 2. GENIE - Detailed somatic pathogenic variant results**
- **Supplementary Table 3. INOVATe – Mean and median age at diagnosis and age at testing of patients with *BRCA1/2* variants**
- **Supplementary Table 4. Variant data from INOVATe**
- **Supplementary Table 5. TCGA Nature 2011 dataset (Study ID ov_tcga_pub)^1^**
- **Supplementary Table 6. Results from AOCS/ICGC data^2^**

**Supplementary Table 1. GENIE - Detailed age at reporting results of patients with somatic pathogenic variants.**

| **Gene** | *BRCA1* | *BRCA2* | *ATM* | *PALB2* | *BRIP1* | *RAD51D* | *NBN* | *BARD1* | *MRE11* | *CHEK2* | *ABRAXAS1* | *CHEK1* |
| --- | --- | --- | --- | --- | --- | --- | --- | --- | --- | --- | --- | --- |
| Number of samples | 58 | 27 | 6 | 1 | 3 | 1 | 3 | 1 | 2 | 2 | 1 | 1 |
| **AGE** |  |  |  |  |  |  |  |  |  |  |  |  |
| Minimum | 38 | 46 | 46 | NA | 62 | NA | 58 | NA | 54 | 52 | NA | NA |
| 25% Percentile | 53 | 60 | 46 | NA | 62 | NA | 58 | NA | 54 | 52 | NA | NA |
| **Median** | 60 | 71 | 61 | 67 | 62 | 64 | 65 | 47 | 60 | 55 | 60 | 40 |
| 75% Percentile | 67 | 79 | 72 | NA | 62 | NA | 67 | NA | 67 | 58 | NA | NA |
| Maximum | 85 | 90 | 72 | NA | 62 | NA | 67 | NA | 67 | 58 | NA | NA |
| Range | 47 | 44 | 26 | NA | 0 | NA | 9 | NA | 13 | 6 | NA | NA |
|  |  |  |  |  |  |  |  |  |  |  |  |  |
| **Mean** | 60 | 69 | 60 | 67 | 62 | 64 | 63 | 47 | 60. | 55 | 60 | 40 |
| Std. Deviation | 10.6 | 12.2 | 12.2 | 0 | 0 | 0 | 4.7 | 0 | 9.2 | 4.2 | 0 | 0 |
| Std. Error of Mean | 1.4 | 2.4 | 5.0 | 0 | 0 | 0 | 2.7 | 0 | 6.5 | 3 | 0 | 0 |

**Supplementary Table 2. GENIE - Detailed somatic pathogenic variant results**

| **Sample**  **ID** | **Patient ID** | **Gene** | **Variant** | **Mutation type** | **Age^#^** | **HGVSg** | **HGVSc** | **Allele Freq (T)** |
| --- | --- | --- | --- | --- | --- | --- | --- | --- |
| GENIE-MSK-P-0045890-T01-IM6 | GENIE-MSK-P-0045890 | *BRCA1* | L22Vfs*7 | Frame_Shift_Del | 40 | 17:g.41276045_41276051del | ENST00000357654.3: c.63_69del | 0.55 |
| GENIE-MSK-P-0042757-T01-IM6 | GENIE-MSK-P-0042757 | *BRCA1* | C27* | Nonsense_Mutation | 46 | 17:g.41267796A>T | ENST00000357654.3: c.81T>A | 0.43 |
| GENIE-MSK-P-0035101-T01-IM6 | GENIE-MSK-P-0035101 | *BRCA1* | K38Nfs*5 | Frame_Shift_Del | 60 | 17:g.41267743_41267764del | ENST00000357654.3: c.113_134del | 0.45 |
| GENIE-MSK-P-0052358-T01-IM6 | GENIE-MSK-P-0052358 | *BRCA1* | K110Rfs*2 | Frame_Shift_Del | 54 | 17:g.41256223_41256251del | ENST00000357654.3: c.329_357del | 0.23 |
| GENIE-MSK-P-0016058-T01-IM6 | GENIE-MSK-P-0016058 | *BRCA1* | S281Afs*17 | Frame_Shift_Del | 76 | 17:g.41246707del | ENST00000357654.3: c.841del | 0.13 |
| GENIE-MSK-P-0052351-T01-IM6 | GENIE-MSK-P-0052351 | *BRCA1* | D295Tfs*3 | Frame_Shift_Del | 56 | 17:g.41246665del | ENST00000357654.3: c.883del | 0.69 |
| GENIE-MSK-P-0033690-T01-IM6 | GENIE-MSK-P-0033690 | *BRCA1* | L393* | Nonsense_Mutation | 52 | 17:g.41246370A>C | ENST00000357654.3: c.1178T>G | 0.37 |
| GENIE-MSK-P-0047390-T01-IM6 | GENIE-MSK-P-0047390 | *BRCA1* | S426* | Nonsense_Mutation | 56 | 17:g.41246271G>C | ENST00000357654.3: c.1277C>G | 0.06 |
| GENIE-MSK-P-0040043-T01-IM6 | GENIE-MSK-P-0040043 | *BRCA1* | K450Nfs*3 | Frame_Shift_Del | 64 | 17:g.41246198del | ENST00000357654.3: c.1350del | 0.72 |
| GENIE-MSK-P-0027262-T01-IM6 | GENIE-MSK-P-0027262 | *BRCA1* | R507Dfs*25 | Frame_Shift_Del | 56 | 17:g.41246029del | ENST00000357654.3: c.1519del | 0.92 |
| GENIE-MSK-P-0040154-T01-IM6 | GENIE-MSK-P-0040154 | *BRCA1* | Q534* | Nonsense_Mutation | 64 | 17:g.41245948G>A | ENST00000357654.3: c.1600C>T | 0.7 |
| GENIE-MSK-P-0052058-T01-IM6 | GENIE-MSK-P-0052058 | *BRCA1* | Q534* | Nonsense_Mutation | 52 | 17:g.41245948G>A | ENST00000357654.3: c.1600C>T | 0.41 |
| GENIE-MSK-P-0044770-T01-IM6 | GENIE-MSK-P-0044770 | *BRCA1* | T549Mfs*19 | Frame_Shift_Del | 67 | 17:g.41245890_41245902del | ENST00000357654.3: c.1646_1658del | 0.38 |
| GENIE-MSK-P-0026504-T01-IM6 | GENIE-MSK-P-0026504 | *BRCA1* | S645Lfs*6 | Frame_Shift_Del | 48 | 17:g.41245615del | ENST00000357654.3: c.1933del | 0.71 |
| GENIE-MSK-P-0059268-T01-IM7 | GENIE-MSK-P-0059268 | *BRCA1* | K65* | Frame_Shift_Ins | 65 | 17:g.41245594_41245595delinsAA | ENST00000357654.3: c.1953_1954delinsTT | 0.58 |
| GENIE-MSK-P-0038779-T01-IM6 | GENIE-MSK-P-0038779 | *BRCA1* | K651_K652delinsN* | Nonsense_Mutation | 68 | 17:g.41245594_41245595delinsAA | ENST00000357654.3: c.1953_1954delinsTT | 0.58 |
| GENIE-MSK-P-0033193-T01-IM6 | GENIE-MSK-P-0033193 | *BRCA1* | E720Nfs*16 | Frame_Shift_Del | 64 | 17:g.41245391del | ENST00000357654.3: c.2157del | 0.85 |
| GENIE-MSK-P-0048171-T01-IM6 | GENIE-MSK-P-0048171 | *BRCA1* | T737Qfs*16 | Frame_Shift_Del | 60 | 17:g.41245339del | ENST00000357654.3: c.2209del | 0.72 |
| GENIE-MSK-P-0055860-T01-IM6 | GENIE-MSK-P-0055860 | *BRCA1* | L752* | Nonsense_Mutation | 79 | 17:g.41245293A>C | ENST00000357654.3: c.2255T>G | 0.7 |
| GENIE-MSK-P-0039468-T01-IM6 | GENIE-MSK-P-0039468 | *BRCA1* | S784Rfs*8 | Frame_Shift_Del | 79 | 17:g.41245198del | ENST00000357654.3: c.2350del | 0.53 |
| GENIE-MSK-P-0056722-T01-IM6 | GENIE-MSK-P-0056722 | *BRCA1* | K830* | Frame_Shift_Ins | 48 | 17:g.41245060_41245061insA | ENST00000357654.3: c.2487dup | 0.62 |
| GENIE-MSK-P-0056197-T01-IM6 | GENIE-MSK-P-0056197 | *BRCA1* | E914* | Nonsense_Mutation | 44 | 17:g.41244808C>A | ENST00000357654.3: c.2740G>T | 0.49 |
| GENIE-MSK-P-0038526-T01-IM6 | GENIE-MSK-P-0038526 | *BRCA1* | E1013Dfs*4 | Frame_Shift_Del | 65 | 17:g.41244508_41244509del | ENST00000357654.3: c.3039_3040del | 0.25 |
| GENIE-MSK-P-0019908-T01-IM6 | GENIE-MSK-P-0019908 | *BRCA1* | M1014Wfs*10 | Frame_Shift_Del | 60 | 17:g.41244508del | ENST00000357654.3: c.3040del | 0.64 |
| GENIE-MSK-P-0025545-T01-IM6 | GENIE-MSK-P-0025545 | *BRCA1* | E1033* | Nonsense_Mutation | 38 | 17:g.41244451C>A | ENST00000357654.3: c.3097G>T | 0.75 |
| GENIE-MSK-P-0051798-T01-IM6 | GENIE-MSK-P-0051798 | *BRCA1* | P1099Lfs*10 | Frame_Shift_Del | U | 17:g.41244252del | ENST00000357654.3: c.3296del | 0.3 |
| GENIE-MSK-P-0038543-T01-IM6 | GENIE-MSK-P-0038543 | *BRCA1* | S1101* | Frame_Shift_Del | 63 | 17:g.41244247_41244248del | ENST00000357654.3: c.3300_3301del | 0.59 |
| GENIE-MSK-P-0019391-T01-IM6 | GENIE-MSK-P-0019391 | *BRCA1* | C1146* | Nonsense_Mutation | 69 | 17:g.41244110A>T | ENST00000357654.3: c.3438T>A | 0.87 |
| GENIE-MSK-P-0003813-T01-IM5 | GENIE-MSK-P-0003813 | *BRCA1* | E1167* | Nonsense_Mutation | 67 | 17:g.41244049C>A | ENST00000357654.3: c.3499G>T | 0.26 |
| GENIE-MSK-P-0034250-T01-IM6 | GENIE-MSK-P-0034250 | *BRCA1* | Q1200* | Nonsense_Mutation | 60 | 17:g.41243950G>A | ENST00000357654.3: c.3598C>T | 0.25 |
| GENIE-MSK-P-0028702-T01-IM6 | GENIE-MSK-P-0028702 | *BRCA1* | R1203* | Nonsense_Mutation | 69 | 17:g.41243941G>A | ENST00000357654.3: c.3607C>T | 0.71 |
| GENIE-MSK-P-0057796-T01-IM6 | GENIE-MSK-P-0057796 | *BRCA1* | E1282Nfs*25 | Frame_Shift_Del | 55 | 17:g.41243704del | ENST00000357654.3: c.3844del | 0.43 |
| GENIE-MSK-P-0021742-T01-IM6 | GENIE-MSK-P-0021742 | *BRCA1* | L1303Sfs*15 | Frame_Shift_Del | 46 | 17:g.41243606_41243640del | ENST00000357654.3: c.3908_3942del | 0.7 |
| GENIE-MSK-P-0038306-T01-IM6 | GENIE-MSK-P-0038306 | *BRCA1* | T1307Lfs*11 | Frame_Shift_Del | 60 | 17:g.41243630del | ENST00000357654.3: c.3918del | 0.54 |
| GENIE-MSK-P-0053402-T01-IM6 | GENIE-MSK-P-0053402 | *BRCA1* | D1314Vfs*4 | Frame_Shift_Del | 64 | 17:g.41243607del | ENST00000357654.3: c.3941del | 0.35 |
| GENIE-MSK-P-0018550-T01-IM6 | GENIE-MSK-P-0018550 | *BRCA1* | N1355Kfs*10 | Frame_Shift_Del | 72 | 17:g.41243480_41243483del | ENST00000357654.3: c.4065_4068del | 0.44 |
| GENIE-MSK-P-0059659-T01-IM7 | GENIE-MSK-P-0059659 | *BRCA1* | Q1388* | Nonsense_Mutation | 51 | 17:g.41242984G>A | ENST00000357654.3: c.4162C>T | 0.36 |
| GENIE-MSK-P-0038096-T01-IM6 | GENIE-MSK-P-0038096 | *BRCA1* | Q1420* | Nonsense_Mutation | 72 | 17:g.41234520G>A | ENST00000357654.3: c.4258C>T | 0.29 |
| GENIE-MSK-P-0050669-T01-IM6 | GENIE-MSK-P-0050669 | *BRCA1* | D1441Efs*21 | Frame_Shift_Ins | 40 | 17:g.41234455_41234456insT | ENST00000357654.3: c.4322dup | 0.62 |
| GENIE-MSK-P-0001911-T01-IM3 | GENIE-MSK-P-0001911 | *BRCA1* | E1446* | Nonsense_Mutation | 56 | 17:g.41234442C>A | ENST00000357654.3: c.4336G>T | 0.44 |
| GENIE-MSK-P-0029393-T01-IM6 | GENIE-MSK-P-0029393 | *BRCA1* | D1475Tfs*30 | Frame_Shift_Del | 64 | 17:g.41228566del | ENST00000357654.3: c.4423del | 0.87 |
| GENIE-MSK-P-0042815-T01-IM6 | GENIE-MSK-P-0042815 | *BRCA1* | N1519Ifs*29 | Frame_Shift_Del | 56 | 17:g.41226467del | ENST00000357654.3: c.4556del | 0.21 |
| GENIE-MSK-P-0040386-T01-IM6 | GENIE-MSK-P-0040386 | *BRCA1* | E1536Sfs*12 | Frame_Shift_Del | 69 | 17:g.41226417del | ENST00000357654.3: c.4606del | 0.6 |
| GENIE-MSK-P-0038583-T01-IM6 | GENIE-MSK-P-0038583 | *BRCA1* | X1559_splice | Splice_Site | 56 | 17:g.41226347C>T | ENST00000357654.3: c.4675+1G>A | 0.54 |
| GENIE-MSK-P-0056529-T01-IM7 | GENIE-MSK-P-0056529 | *BRCA1* | X1559_splice | Splice_Site | 85 | 17:g.41223257T>C | ENST00000357654.3: c.4676-2A>G | 0.43 |
| GENIE-MSK-P-0036348-T01-IM6 | GENIE-MSK-P-0036348 | *BRCA1* | S1569Afs*32 | Frame_Shift_Del | 85 | 17:g.41223227del | ENST00000357654.3: c.4704del | 0.6 |
| GENIE-MSK-P-0033218-T02-IM6 | GENIE-MSK-P-0033218 | *BRCA1* | S1569Afs*32 | Frame_Shift_Del | 50 | 17:g.41223227del | ENST00000357654.3: c.4704del | 0.7 |
| GENIE-MSK-P-0020831-T01-IM6 | GENIE-MSK-P-0020831 | *BRCA1* | K1606* | Nonsense_Mutation | 52 | 17:g.41223115T>A | ENST00000357654.3: c.4816A>T | 0.35 |
| GENIE-MSK-P-0048531-T01-IM6 | GENIE-MSK-P-0048531 | *BRCA1* | E1638* | Nonsense_Mutation | 69 | 17:g.41223019C>A | ENST00000357654.3: c.4912G>T | 0.22 |
| GENIE-MSK-P-0039932-T01-IM6 | GENIE-MSK-P-0039932 | *BRCA1* | X1692_splice | Splice_Site | 60 | 17:g.41219623A>T | ENST00000357654.3: c.5074+2T>A | 0.69 |
| GENIE-MSK-P-0035124-T01-IM6 | GENIE-MSK-P-0035124 | *BRCA1* | X1692_splice | Splice_Site | 59 | 17:g.41215970T>G | ENST00000357654.3: c.5075-2A>C | 0.08 |
| GENIE-MSK-P-0040960-T01-IM6 | GENIE-MSK-P-0040960 | *BRCA1* | R1699W | Missense_Mutation | 46 | 17:g.41215948G>A | ENST00000357654.3: c.5095C>T | 0.38 |
| GENIE-MSK-P-0046282-T01-IM6 | GENIE-MSK-P-0046282 | *BRCA1* | W1712* | Nonsense_Mutation | 73 | 17:g.41215908C>T | ENST00000357654.3: c.5135G>A | 0.6 |
| GENIE-MSK-P-0037128-T01-IM6 | GENIE-MSK-P-0037128 | *BRCA1* | W1712* | Nonsense_Mutation | 56 | 17:g.41215907C>T | ENST00000357654.3: c.5136G>A | 0.32 |
| GENIE-MSK-P-0034907-T01-IM6 | GENIE-MSK-P-0034907 | *BRCA1* | W1718* | Nonsense_Mutation | 62 | 17:g.41215390C>T | ENST00000357654.3: c.5153G>A | 0.76 |
| GENIE-MSK-P-0014391-T01-IM6 | GENIE-MSK-P-0014391 | *BRCA1* | W1782* | Nonsense_Mutation | 62 | 17:g.41201199C>T | ENST00000357654.3: c.5345G>A | 0.18 |
| GENIE-MSK-P-0021359-T01-IM6 | GENIE-MSK-P-0021359 | *BRCA1* | X1803_splice | Splice_Site | 59 | 17:g.41199721C>G | ENST00000357654.3: c.5407-1G>C | 0.07 |
| GENIE-MSK-P-0037160-T01-IM6 | GENIE-MSK-P-0037160 | *BRCA1* | W1815* | Nonsense_Mutation | 67 | 17:g.41199682C>T | ENST00000357654.3: c.5445G>A | 0.88 |
| GENIE-MSK-P-0056448-T01-IM6 | GENIE-MSK-P-0056448 | *BRCA2* | X159_splice | Splice_Site | 76 | 13:g.32900377A>G | ENST00000380152.3: c.476-2A>G | 0.77 |
| GENIE-MSK-P-0028443-T01-IM6 | GENIE-MSK-P-0028443 | *BRCA2* | N272Ifs*5 | Frame_Shift_Del | 65 | 13:g.32906426del | ENST00000380152.3: c.813del | 0.79 |
| GENIE-MSK-P-0001284-T01-IM3 | GENIE-MSK-P-0001284 | *BRCA2* | R329Gfs*20 | Frame_Shift_Del | 56 | 13:g.32906599del | ENST00000380152.3: c.984del | 0.23 |
| GENIE-MSK-P-0030779-T01-IM6 | GENIE-MSK-P-0030779 | *BRCA2* | V545Hfs*11 | Frame_Shift_Del | 48 | 13:g.32907246_32907252del | ENST00000380152.3: c.1633_1639del | 0.51 |
| GENIE-MSK-P-0055215-T01-IM6 | GENIE-MSK-P-0055215 | *BRCA2* | P655Qfs*5 | Frame_Shift_Del | 81 | 13:g.32910455del | ENST00000380152.3: c.1964del | 0.69 |
| GENIE-MSK-P-0037375-T01-IM6 | GENIE-MSK-P-0037375 | *BRCA2* | G800Vfs*10 | Frame_Shift_Del | 68 | 13:g.32910887del | ENST00000380152.3: c.2397del | 0.34 |
| GENIE-MSK-P-0018647-T01-IM6 | GENIE-MSK-P-0018647 | *BRCA2* | Q893Kfs*2 | Frame_Shift_Del | 84 | 13:g.32911168del | ENST00000380152.3: c.2677del | 0.64 |
| GENIE-MSK-P-0059109-T01-IM7 | GENIE-MSK-P-0059109 | *BRCA2* | E953* | Nonsense_Mutation | 84 | 13:g.32911349G>T | ENST00000380152.3: c.2857G>T | 0.51 |
| GENIE-MSK-P-0058853-T01-IM7 | GENIE-MSK-P-0058853 | *BRCA2* | V1068Yfs*9 | Frame_Shift_Del | 62 | 13:g.32911693del | ENST00000380152.3: c.3201del | 0.22 |
| GENIE-MSK-P-0057038-T01-IM6 | GENIE-MSK-P-0057038 | *BRCA2* | S1106Afs*13 | Frame_Shift_Del | 58 | 13:g.32911807del | ENST00000380152.3: c.3315del | 0.54 |
| GENIE-MSK-P-0025454-T01-IM6 | GENIE-MSK-P-0025454 | *BRCA2* | Q1138Rfs*12 | Frame_Shift_Del | 46 | 13:g.32911905del | ENST00000380152.3: c.3413del | 0.61 |
| GENIE-MSK-P-0049075-T01-IM6 | GENIE-MSK-P-0049075 | *BRCA2* | A1170Gfs*5 | Frame_Shift_Del | 71 | 13:g.32912001_32912007del | ENST00000380152.3: c.3509_3515del | 0.41 |
| GENIE-MSK-P-0057733-T01-IM6 | GENIE-MSK-P-0057733 | *BRCA2* | K1286* | Frame_Shift_Del | 65 | 13:g.32912346_32912350del | ENST00000380152.3: c.3856_3860del | 0.96 |
| GENIE-MSK-P-0041985-T01-IM6 | GENIE-MSK-P-0041985 | *BRCA2* | N1344Mfs*30 | Frame_Shift_Del | 47 | 13:g.32912519del | ENST00000380152.3: c.4031del | 0.71 |
| GENIE-MSK-P-0039094-T01-IM6 | GENIE-MSK-P-0039094 | *BRCA2* | D1386Wfs*20 | Frame_Shift_Del | 60 | 13:g.32912648_32912660del | ENST00000380152.3: c.4156_4168del | 0.56 |
| GENIE-MSK-P-0029084-T01-IM6 | GENIE-MSK-P-0029084 | *BRCA2* | C1591Vfs*26 | Frame_Shift_Del | 76 | 13:g.32913263del | ENST00000380152.3: c.4771del | 0.39 |
| GENIE-MSK-P-0036542-T01-IM6 | GENIE-MSK-P-0036542 | *BRCA2* | N1784Kfs*3 | Frame_Shift_Ins | 84 | 13:g.32913836_32913837insA | ENST00000380152.3: c.5351dup | 0.17 |
| GENIE-MSK-P-0018996-T01-IM6 | GENIE-MSK-P-0018996 | *BRCA2* | F1870Kfs*7 | Frame_Shift_Del | 67 | 13:g.32914100_32914114delinsA | ENST00000380152.3: c.5608_5622delinsA | 0.56 |
| GENIE-MSK-P-0053010-T01-IM6 | GENIE-MSK-P-0053010 | *BRCA2* | Q2009Sfs*30 | Frame_Shift_Del | 60 | 13:g.32914513_32914516del | ENST00000380152.3: c.6025_6028del | 0.51 |
| GENIE-MSK-P-0059631-T01-IM7 | GENIE-MSK-P-0059631 | *BRCA2* | Q2163* | Nonsense_Mutation | 83 | 13:g.32914979C>T | ENST00000380152.3: c.6487C>T | 0.22 |
| GENIE-MSK-P-0030052-T01-IM6 | GENIE-MSK-P-0030052 | *BRCA2* | E2198Nfs*4 | Frame_Shift_Del | 58 | 13:g.32915083_32915084del | ENST00000380152.3: c.6591_6592del | 0.35 |
| GENIE-MSK-P-0018997-T01-IM6 | GENIE-MSK-P-0018997 | *BRCA2* | V2423* | Frame_Shift_Del | 73 | 13:g.32929253_32929254del | ENST00000380152.3: c.7267_7268del | 0.08 |
| GENIE-MSK-P-0055169-T02-IM6 | GENIE-MSK-P-0055169 | *BRCA2* | Q2957* | Nonsense_Mutation | 75 | 13:g.32953568C>T | ENST00000380152.3: c.8869C>T | 0.35 |
| GENIE-MSK-P-0039012-T01-IM6 | GENIE-MSK-P-0039012 | *BRCA2* | T3033Nfs*11 | Frame_Shift_Ins | 76 | 13:g.32954022_32954023insA | ENST00000380152.3: c.9097dup | 0.47 |
| GENIE-MSK-P-0060551-T01-IM7 | GENIE-MSK-P-0060551 | *BRCA2* | T3033Nfs*11 | Frame_Shift_Ins | 72 | 13:g.32954022_32954023insA | ENST00000380152.3: c.9097dup | 0.57 |
| GENIE-MSK-P-0046957-T01-IM6 | GENIE-MSK-P-0046957 | *BRCA2* | E1646* | Nonsense_Mutation | 79 | 13:g.32954022_32954023insA | ENST00000380152.3: c.9097dup | 0.57 |
| GENIE-MSK-P-0018944-T01-IM6 | GENIE-MSK-P-0018944 | *BRCA2* | W3106* | Nonsense_Mutation | >89 | 13:g.32968886G>A | ENST00000380152.3: c.9317G>A | 0.76 |
| GENIE-MSK-P-0051273-T01-IM6 | GENIE-MSK-P-0051273 | *ABRAXAS1* | X72_splice | Splice_Site | 60 | 4:g.84393442C>T | ENST00000321945.7: c.216-1G>A | 0.34 |
| GENIE-MSK-P-0041234-T01-IM6 | GENIE-MSK-P-0041234 | *ATM* | X536_splice | Splice_Site | 55 | 11:g.108121800G>A | ENST00000278616.4: c.1607+1G>A | 0.19 |
| GENIE-MSK-P-0047089-T01-IM6 | GENIE-MSK-P-0047089 | *ATM* | V723Lfs*10 | Frame_Shift_Del | 72 | 11:g.108126983_108126996del | ENST00000278616.4: c.2167_2180del | 0.09 |
| GENIE-MSK-P-0031960-T01-IM6 | GENIE-MSK-P-0031960 | *ATM* | P1412Lfs*39 | Frame_Shift_Del | 72 | 11:g.108159827del | ENST00000278616.4: c.4235del | 0.15 |
| GENIE-MSK-P-0054490-T01-IM6 | GENIE-MSK-P-0054490 | *ATM* | A2364Gfs*9 | Frame_Shift_Ins | 47 | 11:g.108198480_108198481insA | ENST00000278616.4: c.7088dup | 0.27 |
| GENIE-MSK-P-0054671-T01-IM6 | GENIE-MSK-P-0054671 | *ATM* | V2774Afs*32 | Frame_Shift_Del | 46 | 11:g.108214001del | ENST00000278616.4: c.8321del | 0.61 |
| GENIE-MSK-P-0017005-T01-IM6 | GENIE-MSK-P-0017005 | *ATM* | X2891_splice | Splice_Site | 68 | 11:g.108224492G>A | ENST00000278616.4: c.8672-1G>A | 0.42 |
| GENIE-MSK-P-0041614-T01-IM6 | GENIE-MSK-P-0041614 | *BARD1* | X72_splice | Splice_Site | 47 | 2:g.215661784C>T | ENST00000260947.4: c.215+1G>A | 0.36 |
| GENIE-MSK-P-0002373-T01-IM3 | GENIE-MSK-P-0002373 | *BRIP1* | X170_splice | Splice_Site | 62 | 17:g.59924582C>G | ENST00000259008.2: c.508-1G>C | 0.19 |
| GENIE-MSK-P-0036872-T01-IM6 | GENIE-MSK-P-0036872 | *BRIP1* | C832W | Missense_Mutation | 62 | 17:g.59770870A>C | ENST00000259008.2: c.2496T>G | 0.78 |
| GENIE-MSK-P-0039583-T01-IM6 | GENIE-MSK-P-0039583 | *BRIP1* | R865W | Missense_Mutation | 62 | 17:g.59763509G>A | ENST00000259008.2: c.2593C>T | 0.15 |
| GENIE-MSK-P-0013630-T01-IM5 | GENIE-MSK-P-0013630 | *CHEK1* | *477Sext*24 | Nonstop_Mutation | 40 | 11:g.125525214G>C | ENST00000428830.2: c.1430G>C | 0.28 |
| GENIE-MSK-P-0003829-T01-IM5 | GENIE-MSK-P-0003829 | *CHEK2* | X282_splice | Splice_Site | 52 | 22:g.29105993C>T | ENST00000328354.6: c.846+1G>A | 0.46 |
| GENIE-MSK-P-0061394-T01-IM7 | GENIE-MSK-P-0061394 | *CHEK2* | F369Lfs*13 | Frame_Shift_Del | 58 | 22:g.29091850del | ENST00000328354.6: c.1107del | 0.39 |
| GENIE-MSK-P-0004175-T01-IM5 | GENIE-MSK-P-0004175 | *MRE11* | X367_splice | Splice_Site | 54 | 11:g.94197379_94197415del | ENST00000323929.3: c.1099-10_1125del | 0.12 |
| GENIE-MSK-P-0001985-T01-IM3 | GENIE-MSK-P-0001985 | *MRE11* | Q629Afs*9 | Frame_Shift_Del | 67 | 11:g.94170383_94170384del | ENST00000323929.3: c.1885_1886del | 0.27 |
| GENIE-MSK-P-0037167-T01-IM6 | GENIE-MSK-P-0037167 | *NBN* | M1? | Translation_Start_Site | 58 | 8:g.90996788A>G | ENST00000265433.3: c.2T>C | 0.26 |
| GENIE-MSK-P-0019234-T01-IM6 | GENIE-MSK-P-0019234 | *NBN* | T226Hfs*5 | Frame_Shift_Del | 65 | 8:g.90983434del | ENST00000265433.3: c.669del | 0.07 |
| GENIE-MSK-P-0021908-T01-IM6 | GENIE-MSK-P-0021908 | *NBN* | L655Yfs*2 | Frame_Shift_Del | 67 | 8:g.90958474del | ENST00000265433.3: c.1964del | 0.08 |
| GENIE-MSK-P-0021524-T01-IM6 | GENIE-MSK-P-0021524 | *PALB2^¶^ (ATM also present)* | W904* | Nonsense_Mutation | 67 | 16:g.23637593C>T | ENST00000261584.4: c.2712G>A | 0.2 |
| GENIE-MSK-P-0055228-T01-IM6 | GENIE-MSK-P-0055228 | *RAD51D* | K235Rfs*13 | Frame_Shift_Del | 64 | 17:g.33430309del | ENST00000345365.6: c.702del | 0.45 |

Age at which sequencing was reported. U=age unknown.

Highlighted in blue are results from patients ≥70 years of age.

Sample ID GENIE-MSK-P-0029393-T01-IM6: Extra HOMDEL of ATM and CHEK1.

Sample ID GENIE-MSK-P-0021524-T01-IM6: Which had a PALB2 nonsense variant also had a missense variant in ATM^¶^  L2452Q (Missense mutation)

Sample ID GENIE-MSK-P-0014391-T01-IM6**:** Which had a BRCA1 nonsense variant also had an additional BRCA1 missense variant V741l which was removed from the analysis as not deleterious

One *BRCA1* missense variant A1708P was not included in this analysis as there is no evidence that it is deleterious.

Sorted by gene and variant position.

**Supplementary Table 3. INOVATe – Mean and median age at diagnosis and age at testing of patients with *BRCA1/2* variants**

| **Age at Diagnosis** | | |
| --- | --- | --- |
| **Gene** | ***BRCA1*** | ***BRCA2*** |
| Number of samples | 15 | 10 |
|  |  |  |
| **Patient Age, Diagnosis** |  |  |
| Minimum | 40 | 56 |
| 25% Percentile | 51 | 63 |
| **Median** | 60 | 68 |
| 75% Percentile | 71 | 70 |
| Maximum | 77 | 76 |
| Range | 37 | 20 |
|  |  |  |
| **Mean** | 60.1 | 67.2 |
| Std. Deviation | 11 | 5.7 |
| Std. Error of Mean | 2.8 | 1.8 |

| **Age at Testing** | | |
| --- | --- | --- |
| **Gene** | ***BRCA1*** | ***BRCA2*** |
| Number of samples | 15 | 10 |
|  |  |  |
| **Patient Age, Testing** |  |  |
| Minimum | 41 | 60 |
| 25% Percentile | 57 | 65 |
| **Median** | 63 | 70 |
| 75% Percentile | 73 | 76 |
| Maximum | 79 | 77 |
| Range | 38 | 17 |
|  |  |  |
| **Mean** | 62.8 | 69.9 |
| Std. Deviation | 11 | 6.0 |
| Std. Error of Mean | 2.9 | 1.9 |

**Supplementary Table 4. Variant data from INOVATe**

| **SAMPLE ID** | **Gene** | **Variant** | **Mutation Type** | **Age at Diagnosis** | **Age at Testing** | **HGVSc** | **Variant Allele Frequency** |
| --- | --- | --- | --- | --- | --- | --- | --- |
| 10089-180667-1000-500-47 | *BRCA1* | p.R71= | Splice_Region | 60 | 67 | NM_007300.3:  c.211A>C | 0.85 |
| 10097-190048-1000-500-51 | *BRCA1* | p.? | Whole gene deletion | 46 | 47 | NM_007294.3:  c.1-?_5592+?del |  |
| 10101-190184-1000-500-56 | *BRCA1* | p.E362Rfs*4 | Frame_Shift_Ins | 56 | 57 | NM_007300.3:  c.1083dupA | 0.58 |
| 12033-160134-1000-500-12 | *BRCA1* | p.R1203* | Nonsense_Mutation | 57 | 58 | NM_007300.3:  c.3607C>T | 0.59 |
| 12043-170244-7020-500-21 | *BRCA1* | p.R1443* | Nonsense_Mutation | 72 | 72 | NM_007300.3:  c.4327C>T | 0.44 |
| 12115-180023-1000-500-40 | *BRCA1* | p.T922Lfs*77 | Frame_Shift_Del | 40 | 41 | NM_007300.3:  c.2764_2767delACAG | 0.66 |
| 12122-180224-1020-500-49 | *BRCA1* | p.N1355Kfs*10 | Frame_Shift_Del | 74 | 75 | NM_007300.3:  c.4065_4068delTCAA | 0.66 |
| 12134-180265-1020-500-39 | *BRCA1* | p.K109Rfs*9 | Frame_Shift_Del | 63 | 63 | NM_007300.3:  c.326_329del | 0.49 |
| 12244-190760-1020-500-77 | *BRCA1* | p.N1355Kfs*10 | Frame_Shift_Del | 51 | 51 | NM_007300.3:  c.4065_4068delTCAA | 0.47 |
| 13018-170372-1020-500-Q01 | *BRCA1* | p.K654Sfs*47 | Frame_Shift_Del | 65 | 70 | NM_007300.3:  c.1961delA | 0.56 |
| 13034-180154-1020-500-49 | *BRCA1* | p.W353* | Nonsense_Mutation | 67 | 73 | NM_007300.3:  c.1058G>A | 0.85 |
| 13038-180461-1020-500-53 | *BRCA1* | p.V757Ffs*8 | Frame_Shift_Del | 77 | 79 | NM_007300.3:  c.2269delG | 0.52 |
| 13084-190538-1020-500-Q02 | *BRCA1* | p.K1627Lfs*18 | Frame_Shift_Del | 55 | 58 | NM_007300.3:  c.4879_4906  delAAAGTTGCAGAATCTGCC  CAGAGTCCAG | 0.33 |
| 16013-190465-1020-500-Q02 | *BRCA1* | p.R350* | Nonsense_Mutation | 71 | 73 | NM_007300.3:  c.1048A>T | 0.58 |
| 18019-190471-1020-500-Q02 | *BRCA1* | p.E1112Dfs*5 | Frame_Shift_Del | 48 | 58 | NM_007300.3:  c.3336delA | 0.84 |
| 10023-170324-1000-500-18 | *BRCA2* | p.E1773Dfs*2 | Frame_Shift_Ins | 63 | 65 | NM_000059.3:  c.5315_5318dup | 0.71 |
| 10052-180321-1000-500-48 | *BRCA2* | p.X2479_splice | Splice_Site | 66 | 67 | NM_000059.3:  c.7436-1G>A | 0.84 |
| 10065-180579-7020-500-Q01 | *BRCA2* | p.A938Pfs*21 | Frame_Shift_Del | 69 | 77 | NM_000059.3: c.2808_2811delACAA | 0.42 |
| 10127-190521-1000-500-62 | *BRCA2* | p.F1421Lfs*27 | Frame_Shift_Del | 74 | 75 | NM_000059.3:  c.4263delT | 0.86 |
| 12039-170243-1020-501-41 | *BRCA2* | p.S2670* | Nonsense_Mutation | 68 | 75 | NM_000059.3:  c.8009C>A | 0.47 |
| 12080-170519-1020-500-44 | *BRCA2* | p.S1882* | Nonsense_Mutation | 56 | 60 | NM_000059.3:  c.5645C>A | 0.69 |
| 12173-180685-1020-500-Q01 | *BRCA2* | p.I2521Nfs*16 | Frame_Shift_Del | 76 | 77 | NM_000059.3: c.7560_7567delAATCTCTCinsTAA | 0.73 |
| 13052-190239-1020-500-Q02 | *BRCA2* | p.Q1931* | Nonsense_Mutation | 63 | 64 | NM_000059.3:  c.5791C>T | 0.47 |
| 13086-200104-1020-500-79 | *BRCA2* | p.Y748* | Frame_Shift_Del | 69 | 70 | NM_000059.3:  c.2244_2245delCA | 0.58 |
| 17002-200133-1020-500-83 | *BRCA2* | p.S1882* | Nonsense_Mutation | 68 | 69 | NM_000059.3:  c.5645C>A | 0.20 |

Highlighted in blue are results from patients ≥70 years of age at time of testing.

**Supplementary Table 5. TCGA Nature 2011 dataset (Study ID ov_tcga_pub) -** Results from the 27 patients with somatic mutations in the 13 HRD genes^1^

| **Patient ID** | **Sample ID** | **Somatic PV** | **Protein Change** | **Mutation Type** | **Age at diagnosis#** |
| --- | --- | --- | --- | --- | --- |
| TCGA-04-1331 | TCGA-04-1331-01 | *BRCA2* | *C711** | Nonsense | 79 |
| TCGA-04-1357 | TCGA-04-1357-01 | *BRCA1* | *Q1538** | Nonsense | 52 |
| TCGA-09-2050 | TCGA-09-2050-01 | *BRCA2* | *S1882** | Nonsense | 65 |
| TCGA-13-0726 | TCGA-13-0726-01 | *ATM** | *D1208H* | missense | 55 |
| TCGA-13-0730 | TCGA-13-0730-01 | *BRCA1* | *R1835** | Nonsense | 71 |
| TCGA-13-0761 | TCGA-13-0761-01 | *BRCA1* | *X1495_splice* | splice | 51 |
| TCGA-13-0792 | TCGA-13-0792-01 | *BRCA2** | *E1143D* | missense | 40 |
| TCGA-13-0804 | TCGA-13-0804-01 | *BRCA1* | *C47W* | Missense | 74 |
| TCGA-13-0885 | TCGA-13-0885-01 | *BRCA2* | *K1406Nfs*3* | FS del | 70 |
| TCGA-13-0890 | TCGA-13-0890-01 | *BRCA2* | *S1230Lfs*9* | FS del | 56 |
| TCGA-13-1481 | TCGA-13-1481-01 | *BRCA2* | *S2697Kfs*31* | FS del | 76 |
| TCGA-13-1489 | TCGA-13-1489-01 | *BRCA1* | *N1265Kfs*4* | FS ins | 70 |
| TCGA-13-1501 | TCGA-13-1501-01 | *ATM** | *Y2954C* | missense | 50 |
| TCGA-23-1026 | TCGA-23-1026-01 | *BRCA1* | *G813Dfs*2* | FS del | 45 |
| TCGA-23-1030 | TCGA-23-1030-01 | *BRCA2** | *T1354M* | missense | 64 |
| TCGA-23-1120 | TCGA-23-1120-01 | *BRCA2* | *P3278Lfs*35* | FS Del | 60 |
| TCGA-24-0980 | TCGA-24-0980-01 | *ATM** | *D2507N* | missense | 53 |
| TCGA-24-1103 | TCGA-24-1103-01 | *BRCA2* | *K1638E* | missense | 50 |
| TCGA-24-1466 | TCGA-24-1466-01 | *PALB2* | *K486E* | missense | 74 |
| TCGA-24-1555 | TCGA-24-1555-01 | *BRCA2* | *P2608Qfs*40* | FS del | 50 |
| TCGA-24-1562 | TCGA-24-1562-01 | *CHEK2* | *R346H* | missense | 67 |
| TCGA-24-2035 | TCGA-24-2035-01 | *BRCA1* | *G1710Efs*4* | FS del | 65 |
| TCGA-25-1625 | TCGA-25-1625-01 | *BRCA1* | *E116** | nonsense | 66 |
| TCGA-25-1630 | TCGA-25-1630-01 | *BRCA1* | *A521Qfs*11* | FS del | 73 |
| TCGA-25-1632 | TCGA-25-1632-01 | *BRCA1* | *S1217Rfs*21* | FS ins | 68 |
| TCGA-25-2399 | TCGA-25-2399-01 | *ATM* | *Q2414** | nonsense | 80 |
| TCGA-29-2427 | TCGA-29-2427-01 | *BRCA1* | *L431** | Nonsense | 60 |

All samples are High Grade Serous Ovarian Cancer. Highlighted in blue are results from patients ≥70 years of age.

**Supplementary Table 6. Results from AOCS/ICGC data^2^**

| **Study ID** | **Sample ID** | **Age#** | **HGSC specimen type** | **Collection point** | **Gene** | **Genomic position (chr:start-end)** | **Reference allele** | **Variant allele** | **Exon** | **cDNA change** | **Protein effect^*^** | **Mutation type^*^** |
| --- | --- | --- | --- | --- | --- | --- | --- | --- | --- | --- | --- | --- |
| AOCS-092 | AOCS-092-1 | 68 | Tumour | Primary | *BRCA1* | 17:40487648-41691782 | 1.20 Mb del | - | Whole gene | c.-414514_*710047del | p.? | Deletion (1.20 Mb) |
| AOCS-130 | AOCS-130-1 | 65 | Tumour | Primary | *BRCA1* | 17:41251894-41251894 | C | A | 7 | c.445G>T | p.Glu149* | Nonsense |
| AOCS-086 | AOCS-086-1 | 64 | Tumour | Primary | *BRCA1* | 17:41244022-41244022 | C | - | 10 | c.3526delG | p.Val1176Phefs*34 | Frameshift |
| AOCS-079 | AOCS-079-1 | 72 | Tumour | Primary | *BRCA1* | 17:41243758-41243774 | TCAATGATAATAAATTC | - | 10 | c.3774_3790del | p.Asn1259Glufs*2 | Frameshift |
| AOCS-171 | AOCS-171-1 | 52 | Tumour | Primary | *BRCA1* | 17:41228590-41228590 | G | A | 13 | c.4399C>T | p.Gln1467* | Nonsense |
| AOCS-152 | AOCS-152-1 | 73 | Tumour | Primary | *BRCA1* | 17:41201164-41201164 | C | A | 21 | c.5380G>T | p.Glu1794* | Nonsense |
| AOCS-155^¶^ | AOCS-155-13 |  | Ascites | Recurrence | *BRCA2* | 13:32890600-32890600 | G | A | 2 | c.3G>A | p.? | Missense |
| AOCS-149 | AOCS-149-1 | 56 | Tumour | Primary | *BRCA2* | 13:32911862-32911862 | C | T | 11 | c.3370C>T | p.Gln1124* | Nonsense |
| AOCS-063 | AOCS-063-1 | 62 | Tumour | Primary | *BRCA2* | 13:32912849-32912849 | A | T | 11 | c.4357A>T | p.Lys1453* | Nonsense |
| AOCS-122 | AOCS-122-1 | 59 | Tumour | Primary | *BRCA2* | 13:32914838-32914850 | CACTGTGTAAACT | - | 11 | c.6346_6358del | p.His2116Glnfs*17 | Frameshift |

Highlighted in blue are results from patients ≥70 years of age.

*Variant nomenclature according to Human Genome Variation Society (HGVS) guidelines (http://www.hgvs.org/mutnomen/).

**References – Supplementary Material**

1. Bell D, Berchuck A, Birrer M, et al. Integrated genomic analyses of ovarian carcinoma. *Nature*. 2011/06/01 2011;474(7353):609-615. doi:10.1038/nature10166

2. Patch A-M, Christie EL, Etemadmoghadam D, et al. Whole–genome characterization of chemoresistant ovarian cancer. *Nature*. 2015/05/01 2015;521(7553):489-494. doi:10.1038/nature14410
